# Supplementary material for: Mitogen-Activated Protein Kinase Kinase Kinase 1 Overexpression Disrupts Development of the Ocular Surface Epithelium
Source: Cells. 2025 Jun 13;14(12):894. doi: 10.3390/cells14120894 (PMC12190364; doi:10.3390/cells14120894)
Supplement: Supplementary file 1 [file cells-14-00894-s001.zip › cells-3645547-supplementary.pdf]

Supplementary information

Table S1 Primers for genotyping

Name	Forward 5'-3'	Reverse 5'-3'
5’ external	(P2) ACTCCATATATGGGCTATGAACTAATGACC	(P3) CAATGCTGAAATGTCCCACCAGCC
3’ external	(P4) TGGATTGAGAGACGTTGCACTCAGGTG	(P5) GTTTGCTCCCAAACGGAGTCATTCC
TG allele	(P1) CATACCAGACAAAGGGTGTGACTG	(P2) ACTCCATATATGGGCTATGAACTAATGACC
WT allele	(P1) CATACCAGACAAAGGGTGTGACTG	(P6) GATGGTGTGTTGGGACCTCAGAG

Table S2 chemicals and reagents

Name	Company	Cat. No
Paraformaldehyde (96%)	Alfa Aesar	A11313.36
Mounting Medium Xylene	Fisher Health Care, Pittsburgh	245-691
Penicillin-Streptomycin	Cytiva	SV30010
Keratinocyte-Serum Free Medium (-) calcium chloride (KSFM-Ca)	Gibco	10725-018
Harris Hematoxylin Stain	Azer Scientific, Morgantown, PA	ES701
Eosin-Y Alcoholic Stain	Azer Scientific, Morgantown, PA	ES709
TrypLE^{TM} Express	Gibco	12604-021
Triton X-100	Sigma	9036-19-5
Bovine Serum Albumin	Sigma	9048-46-8
Hoechst 33342	Sigma	B2261
Dimethyl sulfoxide (DMSO)	Sigma	D2650
Blasticidin	Thermo Fisher Scientific	R21001

Table S3 Antibodies

Target protein	Host	Company	Dilution ratio	Cat. No
V5	Rabbit	Cell Signaling Technologies	1:1000	13202S
MAP3K1	Rabbit	Made in house	1:500	
Phosphor-JNK	Rabbit	Cell Signaling Technologies	1:1000	4668S
Phosphor-ERK	Rabbit	Cell Signaling Technologies	1:1000	9101S
Phosphor-c-Jun	Rabbit	Cell Signaling Technologies	1:1000	2361S
Phosphor-p38	Rabbit	Cell Signaling Technologies	1:1000	4631S
β-actin	Mouse	Sigma	1:5000	A1978
E-cadherin	Mouse	BD Biosciences	1:100	610182
Keratin 1	Rabbit	BioLegend	1:100	905602
Keratin 10	Rabbit	BioLegend	1:100	905404
Keratin 12	Rabbit	Invitrogen	1:100	MA5-42701
Keratin 14	Rabbit	Invitrogen	1:100	PA5-28002
α-smooth muscle actin	Rabbit	Abcam	1:100	ab5694
ZO-1	Rabbit	Invitrogen	1:100	40-2200
Anti-mouse IgG	Goat	Invitrogen	1:300	A11004
Anti-rabbit IgG	Goat	Invitrogen	1:300	A11034

Anti-mouse HRP	Goat	PIERCE	1:2000	1858413
Anti-rabbit HRP	Goat	PIERCE	1:2000	1858415

Table S4 Lentiviral particles for reporter cells

Cat#	Product name	Promoter	Reporter	Titration
LTV-0011-4S	TCF/LEF-TAL-BSD	EF1a	Firefly Luc	$2x10^6$(TU)
LTV-0019-4S	AP1-TAL-BSD	EF1a	Firefly Luc	$2x10^6$(TU)
LTV-0010-4S	NOTCH-TAL-BSD	EF1a	Firefly Luc	$2x10^6$(TU)
LTV-0075-4S	BRE-TAL-BSD	EF1a	Firefly Luc	$2x10^6$(TU)
LTV-0028-4S	SRE-TAL-BSD	EF1a	Firefly Luc	$2x10^6$(TU)
